# Decreased Poly(ADP-Ribose) Polymerase 1 Expression Attenuates Glucose Oxidase-Induced Damage in Rat Cochlear Marginal Strial Cells

**DOI:** 10.1007/s12035-015-9469-7

**Published:** 2015-11-02

**Authors:** Yuanyuan Zhang, Yang Yang, Zhen Xie, Wenqi Zuo, Hongyan Jiang, Xueyan Zhao, Yu Sun, Weijia Kong

**Affiliations:** 1 Department of Otorhinolaryngology, Union Hospital, Tongji Medical College, Huazhong University of Science and Technology, 1277 Jiefang Avenue, Wuhan, 430022 China; 2 Department of Otolaryngology—Head and Neck Surgery, Renmin Hospital, Wuhan University, 238 Jiefang Road, Wuhan, 430060 China

**Keywords:** Poly(ADP-ribose) polymerase 1, Marginal cell, Hearing loss, Oxidative stress, Mitochondria, Apoptosis

## Abstract

Oxidative damage to the inner ear is responsible for several types of sensorineural deafness. Cochlear stria marginal cells (MCs) are thought to be vulnerable to such oxidative stress. Activated poly(ADP-ribose) polymerase 1 (PARP1) has been implicated in several diseases, but the effect of PARP1 on MCs subjected to oxidative stress remains elusive. In this study, we established an in vitro cellular oxidative stress model using glucose oxidase (GO) and attempted to explore the role that PARP1 plays in the oxidative damage of MCs. In this study, PARP1 and poly-ADP-ribose (PAR) were highly expressed in GO-treated MCs, and this was accompanied by loss of MC viability, excessive generation of reactive oxygen species (ROS), collapse of mitochondria membrane potential (ΔΨm), and redistribution of the mitochondrial downstream pathway-related molecules Bax and cytochrome c, eventually causing MC death. These effects were almost completely counteracted by suppressing PARP1 expression with small interfering RNA (siRNA). We also found that caspase-3 activation was a downstream event of PARP activation and that apoptosis of MCs was suppressed, although not completely, by pretreatment with the pan-caspase inhibitor z-VAD-fmk. The suppression was less than that when PARP1 expression was inhibited. We conclude that GO treatment induces activation of PARP1, which causes MC damage via mitochondrial mediation. PARP1 plays a pivotal role in GO-induced MC death, at least in part, via the caspase-3 cascade. Our study might provide a new cellular and molecular approach for the treatment of oxidative stress-related sensorineural deafness.

## Introduction

Oxidative damage to the inner ear is responsible for several types of sensorineural hearing loss, including presbycusis, hereditary deafness, noise deafness, and toxicity deafness. The damage causes reduced endocochlear potential (EP), inner ear reactive oxygen species (ROS) accumulation, and cell apoptosis, all of which eventually result in structural changes and functional impairment [1–3]. ROS derived from hydrogen peroxide (H_2_O_2_), superoxide (O_2_), and peroxynitrite (ONOO) are highly reactive and can lead to cell injury [4].

Glucose oxidase (GO) is a steady H_2_O_2_ generator; at a concentration of 10 mU/ml, it can generate H_2_O_2_ continuously at a rate of 1.0–2.4 μM/min for up to 24 h in the presence of 5 mM glucose [5]. Because glucose oxidase catalyzes the oxidation of d-glucose in vitro, high glucose concentrations generate ROS as a result of glucose auto-oxidation. Chang found that glucose oxidase and exogenous H_2_O_2_ have similar biological effects in that both induced apoptosis in HepG2 cells in vitro [5]. Based on these findings, GO was used to induce oxidative stress in our research.

It is well known that mitochondrial DNA (mtDNA) is a biologically important source of adenosine triphosphate (ATP) and that it is located in the vicinity of the respiratory chain, the main site where endogenous ROS are produced. Thus, elevated ROS can damage mitochondrial DNA, which may lead to severe consequences, including impairment of the respiratory chain and decreased ATP synthesis, collapse of mitochondrial membrane potential (ΔΨm), and even cell apoptosis [5, 6]. Because stria marginal cells (MCs) harbor much more mitochondria than other cells in the cochlea, they are particularly vulnerable to ROS attack [7]. Furthermore, MCs are believed to be extremely sensitive to oxidative damage caused by senility and noise [8, 9]. However, the molecular mechanism of oxidative stress-induced apoptosis in marginal cells is not very clear. A better understanding of the cellular and molecular mechanisms associated with oxidative stress-related strial marginal cell degeneration and declines in regenerative capacity is required to identify and explore potential interventional strategies for the prevention and treatment of oxidative stress-related hearing loss.

Poly(ADP-ribose) polymerase 1 (PARP1), a post-translational modification enzyme found mainly in the nucleus but also in a few extranuclear sites of most eukaryotic cells, is called the genome guardian [10]. PARP could be activated when mtDNA is damaged [11], and it catalyzes the hydrolysis of NAD+ into nicotinamide and ADP-ribose, the latter of which is used to synthesize poly-ADP-ribose (PAR) [12], the activated form of PARP1 [13–16]. To prevent excessive NAD+ consumption and energy exhaustion, PARP is specifically disassembled into 24- and 89-kDa fragments by activated caspase-3, and the cells then enter the classic caspase-dependent apoptosis process [17, 18]. The fragments of PARP1 resulting from degradation by caspases are seen as an indicator of apoptosis [19, 20]. However, overactivated PARP1 could eventually lead to the exhaustion of cellular NAD and ATP and cell necrosis [21]. Thus, PARP1 is a key mediator of cell death.

Activated PARP1 has been implicated in several disease models [22–25]. However, the effect of PARP1 on marginal cells subjected to oxidative damage remains elusive. In this study, a PARP1 small interfering RNA (siRNA)-specific adenoviral vector was employed to examine the role that PARP1 plays in the GO-induced oxidative damage of MCs.

## Materials and Methods

### Culture and Identification of Rat MCs

MCs were primarily cultured following the procedures established by our laboratory [26]. Cytokeratin-18 (CK18), a previously established marker of MCs [27, 28], was detected by immunofluorescence. MCs were seeded onto coverslips precoated with poly-l-lysine (Sigma-Aldrich, St. Louis, MO, USA). After the cells fused to form a monolayer, the culture medium was discarded. Then, the samples were washed in cold phosphate-buffered saline (PBS) and fixed with 4 % formalin for 15 min at room temperature. After three more washes with PBS, MCs were permeabilized in 0.3 % Triton TM X-100 (Sigma-Aldrich, St. Louis, MO, USA) for 20 min and then washed three times with PBS. The cells were blocked for 10 min with a 5 % bovine serum albumin (BSA) and then incubated overnight at 4 °C with a primary antibody against CK18 (1:50, Abcam, Cambridge, MA, USA). The following day, the cells were stained with anti-rabbit IgG (1:100) for 30 min at room temperature and then washed three times with PBS. The cells were washed and stained with 4′,6-diamidino-2-phenylindole (DAPI, Sigma-Aldrich, St. Louis, MO, USA) at room temperature for 3 min to identify the nuclei. Afterward, the slides were mounted and visualized under a fluorescence electron microscope.

### GO Exposure

We measured cellular activity by detecting formazan transformed from WST-8 in active cells using a cell counting kit (CCK-8/WST-8, Dojindo Laboratories, Kumamoto, Japan). Briefly, cells were resuspended once the MCs fused to form a monolayer. The MCs were then seeded into 96-well plates, allowed to adhere to the plate, and then treated with different concentrations of GO for 24 h. Each concentration was tested in triplicate. Then, 20 μl of a CCK-8 test solution was added to each well, with 0 mU/ml GO serving as the control group and wells without cells serving as blanks. The absorbance was determined at 450 nm after incubation for 1.5 h at 37 °C. In the CCK-8 assay, methyl thiazolyl tetrazolium WST-8 is cleaved to soluble formazan by active cells, and the corresponding optical density (OD) value is indicative of the cellular activities of MCs. The activity of cells × % = (OD_experimental_ − OD_medium blank_)/(OD_control_ − OD_medium blank_) × 100 %.

### Transfection of MCs with Adenovirus

Once MCs fused to form a monolayer, they were cultured with EpiCM-animal medium containing the Ad-PARP1-RNAi recombinant virus (GeneChem, Shanghai, China) for 4 h. Afterward, the MCs were treated with 20 mU/ml GO for 24 h and then harvested. As a mock-infected group, MCs were also infected with non-specific siRNA. Infection efficiency was assessed in terms of PARP1 expression as measured by reverse transcription-polymerase chain reaction (RT-PCR) and western blotting (described below).

### Grouping

MCs were randomly divided into six groups: (1) control (untreated MCs), (2) GO (MCs were treated with 20 mU/ml GO for 24 h), (3) GO+ Ad-PARP1-RNAi (after infection with Ad-PARP1-RNAi, MCs were treated with 20 mU/ml GO for 24 h), (4) GO + mock (after infection with Ad-mock-RNAi, MCs were treated with 20 mU/ml GO for 24 h), (5) GO + z-VAD-fmk (after treatment with 50 μmol/l z-VAD-fmk for 30 min, MCs were treated with 20 mU/ml GO for 24 h), and (6) z-VAD-fmk (MCs treated with 50 μmol/l z-VAD-fmk for 30 min).

### Evaluation of Apoptosis by Flow Cytometry and DAPI Nuclear Staining

Annexin V-FITC/propidium iodide (PI) double labeling was used to evaluate the rate of MC apoptosis. Briefly, after each treatment, cells were rinsed twice with cold PBS and incubated with trypsin until 80 % of the cells were detached. Cell suspensions were collected and centrifuged at 1000 rpm for 5 min. The supernatant was discarded, and cold PBS was used to wash the pellets two times. The cells (1 × 10^5^) were resuspended in 100 μl of 1× binding buffer containing 5 μl annexin V-FITC and 10 μl PI, incubated for 15 min at room temperature in the dark, and then (BD Biosciences, Carlsbad, CA) analyzed by flow cytometry. After DAPI nuclear staining (as described below), the cells were monitored under a fluorescence microscope for nuclear change.

### Detection of Intracellular ROS Content and Distribution by Flow Cytometry and Laser Confocal Microscopy

The intracellular ROS level was detected using DCFH-DA (Beyotime, Shanghai, China), an oxidation-sensitive fluorescence probe. Cells were seeded into six-well plates (1 × 10^5^ cells/well) and treated in terms of grouping (Fig. 4) for the indicated amount of time. They were then cultured with DCFH-DA (10 μM) for 30 min at room temperature and examined by flow cytometry. The distribution of ROS was detected by co-culturing cells with MitoTracker Red CMXRos (a mitochondria-selective dye; Invitrogen, Carlsbad, CA, USA) and DCFH-DA and then visualizing them by fluorescence microscopy.

Determination of ΔΨm was performed using a multifunctional microplate reader and fluorescence microscopy. Changes in ΔΨm were detected using the Mito-ID membrane potential cytotoxicity kit (ENZO Life Sciences, NY, USA) according to the manufacturer’s instructions. Briefly, after treatment, the cells in each group were loaded with MitoTracker at 37 °C for 30 min. The fluorescence signal (Ex = 485 nm/Em = 590 nm) of ΔΨm was read on a plate reader (BioTek® Synergy 2 Multi-Mode microplate reader, USA) and visualized under a fluorescence microscope.

### Immunofluorescence and Confocal Microscopy

We detected the distribution of PARP1, cytochrome c, and Bax in the MCs by immunofluorescence and confocal microscopy (LSCM, Nikon, Tokyo, Japan). MCs were first seeded onto polylysine-treated coverslips. After the cells fused to form a monolayer, they were treated with 300 nM MitoTracker and then incubated at 37 °C for 45 min. Afterward, the cells were stained with primary antibodies overnight at 4 °C (anti-PARP1 1:50, anti-Bax 1:25, Santa Cruz, CA, USA; anti-CytC 1:100, Invitrogen, Carlsbad, CA, USA). After the specimens were incubated with the corresponding secondary fluorescent antibodies for 30 min, the nuclei were labeled with DAPI for 3 min at room temperature. The MCs were then mounted and observed under an LSCM. The laser beam was set at 488 nm for the FITC-conjugated antibody, at 555 nm for MitoTracker, and at 405 nm for DAPI. Images were acquired simultaneously in the green, red, and blue channels. Images were imported into the Corel Draw X5 software package (Corel, Canada), and the images of representative cells from each well were recorded.

### Measurement of Cytochrome C and Bax in the Mitochondrial/Cytosol

A mitochondria/cytosol isolation kit was used to separate the mitochondria and cytosol of MCs according to the manufacturer’s protocol (BioVision, San Francisco, USA). After treatment with GO (20 μM/ml, 24 h) or pretreatment with Adr for 24 h, cells (5.0 × 10^7^) were collected and suspended in 1 ml of isolation buffer containing protease inhibitors and lysed on ice for 10 min. After mechanical homogenization with a Dunce grinder, a mixture containing unbroken cells, debris, and nuclei was separated by centrifugation at 800×*g* for 10 min at 4 °C. The supernatants were centrifuged at 10,000×*g* for 30 min at 4 °C to obtain the cytosolic fraction (supernatant). The pellets were resuspended in 100 μl Mitochondrial Extraction Buffer Mix containing DTT and protease inhibitors, vortexed for 10 s, and then saved as the mitochondrial fraction. The mitochondria and cytosol were used for detection of cytochrome c and Bax by western blotting.

### Determination of Caspase-3 Activity

Because activated caspase-3 plays a critical role in the final classical pathway in caspase-dependent apoptosis, the activity of caspase-3 was determined using a chemical self-illumination technique that employs Ac-DEVD-AFC (ENZO Life Sciences, USA), a specific fluorogenic substrate for caspase-3. The activity of caspase-3 was determined on a multifunctional microplate reader equipped with a 400-nm excitation filter and a 505-nm emission filter. All assays were carried out in triplicate, and the results were expressed as fold change relative to the control group.

### Western Blotting

MCs seeded in six-well plates (3.0 × 10^5^/well) were pretreated with a virus for 48 h and then treated with GO (20 mU/ml, 24 h). The cells were harvested and homogenized in RIPA lysis buffer (Beyotime, Shanghai, China) containing 1 mM phenylmethylsulfonyl fluoride. The homogenate was centrifuged and the supernatant was collected. Protein concentration was determined using a BCA kit. Cell lysates were subjected to 12 % SDS-polyacrylamide gel electrophoresis (PAGE), followed by protein transfer to a PVDF membrane and incubation with anti-PARP1, anti-Bax (Santa Cruz, CA, USA), anti-PAR (ENZO Life Sciences, USA), anti-CytC (Invitrogen, CA, USA), anti-cleaved caspase-3 (Abcam, Cambridge, UK), and β-actin (Cell Signaling Technology, MA, USA) antibodies. Immunoblots were probed with horseradish peroxidase-conjugated secondary antibodies and visualized using an enhanced chemiluminescent reagent (Millipore, MA, USA). The band intensity was determined by densitometry using a ChemiDoc XRS (Bio-Rad, USA) and normalized to β-actin. The change in protein levels was expressed relative to the control.

### RNA Isolation and Real-Time PCR

Following treatment, messenger ribonucleic acid (mRNA) was isolated from rat MCs using Trizol® reagent (Invitrogen, Carlsbad, CA, USA) according to the manufacturer’s instructions. RNA concentration was measured in terms of the OD_260_/_280_ ratio. mRNA was reverse transcribed into complementary deoxyribonucleic acid (cDNA) using a high-capacity cDNA reverse transcription kit (Applied Biosystems, CA, USA). Real-time PCR was performed in a final volume of 10 μl using Power SYBR Green PCR Master Mix reagents (Applied Biosystems, CA, USA) and run on an ABI Prism 7300 Real-Time PCR System (Applied Biosystems, CA, USA). Each sample was analyzed in triplicate, and glyceraldehyde-3-phosphate dehydrogenase (GAPDH) was used as an internal control. The primer pairs used for PARP1 and GAPDH were as follows: PARP1 forward, 5′-GAGTGGGCACAGTT-ATCGGC-3′; PARP1 reverse, 5′-CCAGGC ATTTCCAGTCTTCTCT-3′; GAPDH forward, 5′-TT CAACGGCACAGT CAAGG-3′; PARP1 reverse, 5′-CTCAGCACCAGCATCACC-3′. The amplification conditions were as follows: 95 °C for 30 s, followed by 40 cycles of denaturation at 95 °C for 30 s, annealing at 60 °C for 60 s, and extension at 95 °C for 5 s. Changes in mRNA levels were corrected against GAPDH using the 2-ΔΔCT method.

### Statistical Analysis

The results were expressed as the mean ± SEM of the three measurements. The statistical analyses were conducted using the SPSS 19.0 software package (IBM, USA). One-way ANOVA and a two-tailed independent-sample *t* test were used for the evaluation of the differences between the treatment and control groups. Differences with a *P* ≤ 0.05 were considered to be significant. The western blotting images were quantitatively assessed by employing ImageJ software (NIH, USA). The chi-square test was used to assess the variation in apoptosis rates between groups, and the corrected *P* value (*P* **′** = *P*/[*k*(*k* − 1)/2]t1) was employed for multiple comparisons.

## Results

### Primary Culture and Identification of MCs

**Fig. 1:**
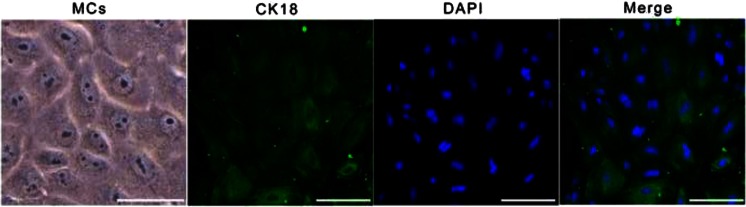
The morphology and identification of MCs. The cultured primary cells with clear boundaries present with a “cobblestone-like” appearance. Immunofluorescence of CK18 (*green*) expressed in the cytoplasm; nuclei are stained *blue*

Figure 1 shows that the MCs present a pleomorphic growth pattern, with a clear boundary. MCs were closely connected and grew into a monolayer, assuming a “cobblestone-like” appearance. Fluorescent signals indicative of CK18 (a biomarker of the epithelium) were strong in the cultured cells.

### Expression of PARP1 in MCs

**Fig. 2:**
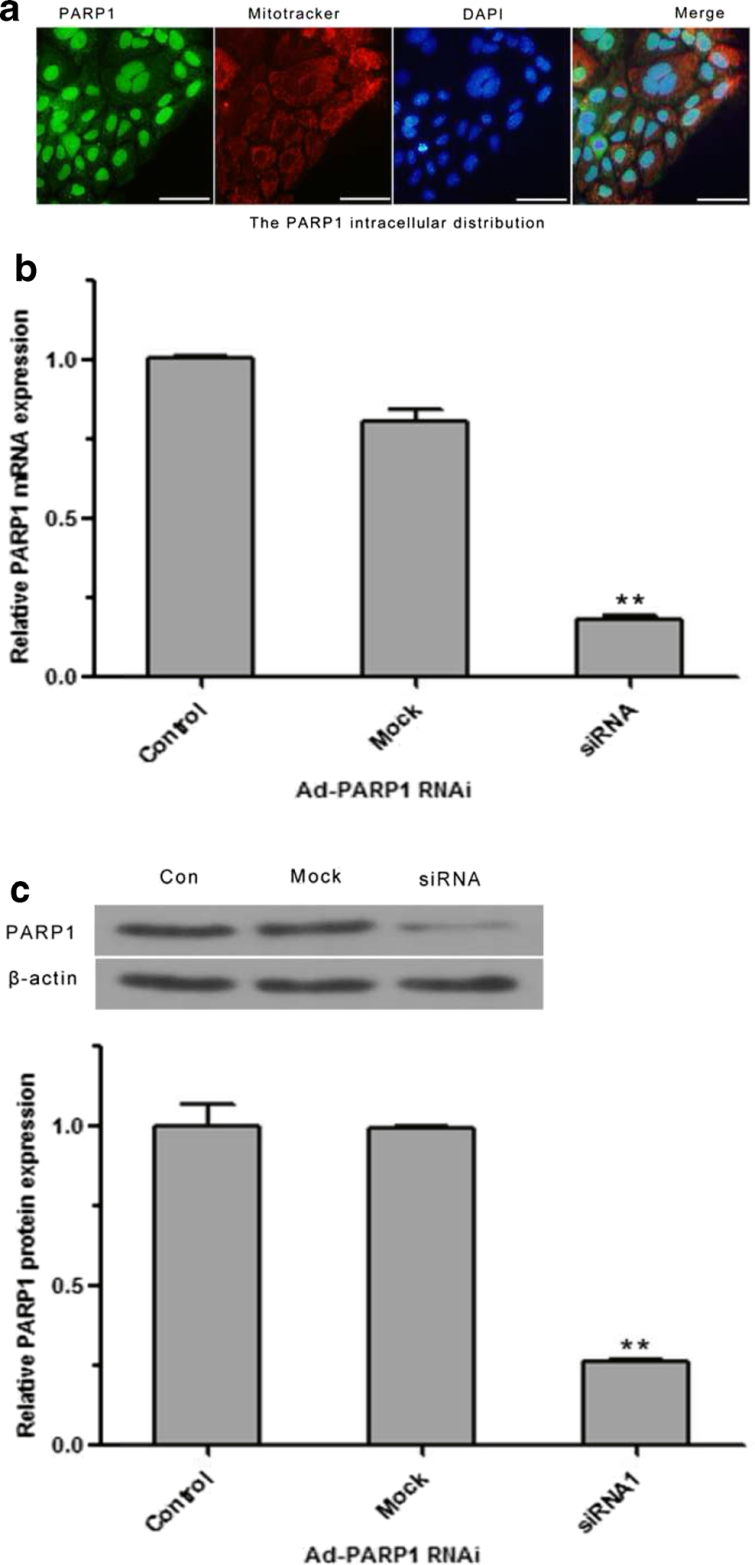
The expression of PARP1 in MCs. **a** PARP1 produces green fluorescence which was mainly found in the nuclei. A few extranuclear PARP1 signals overlapped with the red fluorescence marking the mitochondria. **b**, **c** The changes in PARP1 mRNA and protein expression after cells were infected with Ad-PARP1-RNAi recombinant virus or non-specific siRNA. The data are presented as the mean ± SEM of three separate experiments. **P < 0.01 vs. the control group. *Scale bar* = 50 μm

As shown in Fig. 2a, PARP1 was mainly expressed in nuclei, and a few signals overlapped with the fluorescence of mitochondria. RT-PCR and western blotting showed that after MCs were infected with the Ad-PARP1-RNAi recombinant adenoviruses, PARP1 expression was dramatically decreased at both the mRNA and protein levels when compared to the control and mock-infected groups (Fig. 2b, c).

### Effect of GO Treatment on MC Viability and PARP1 Activity

**Fig. 3:**
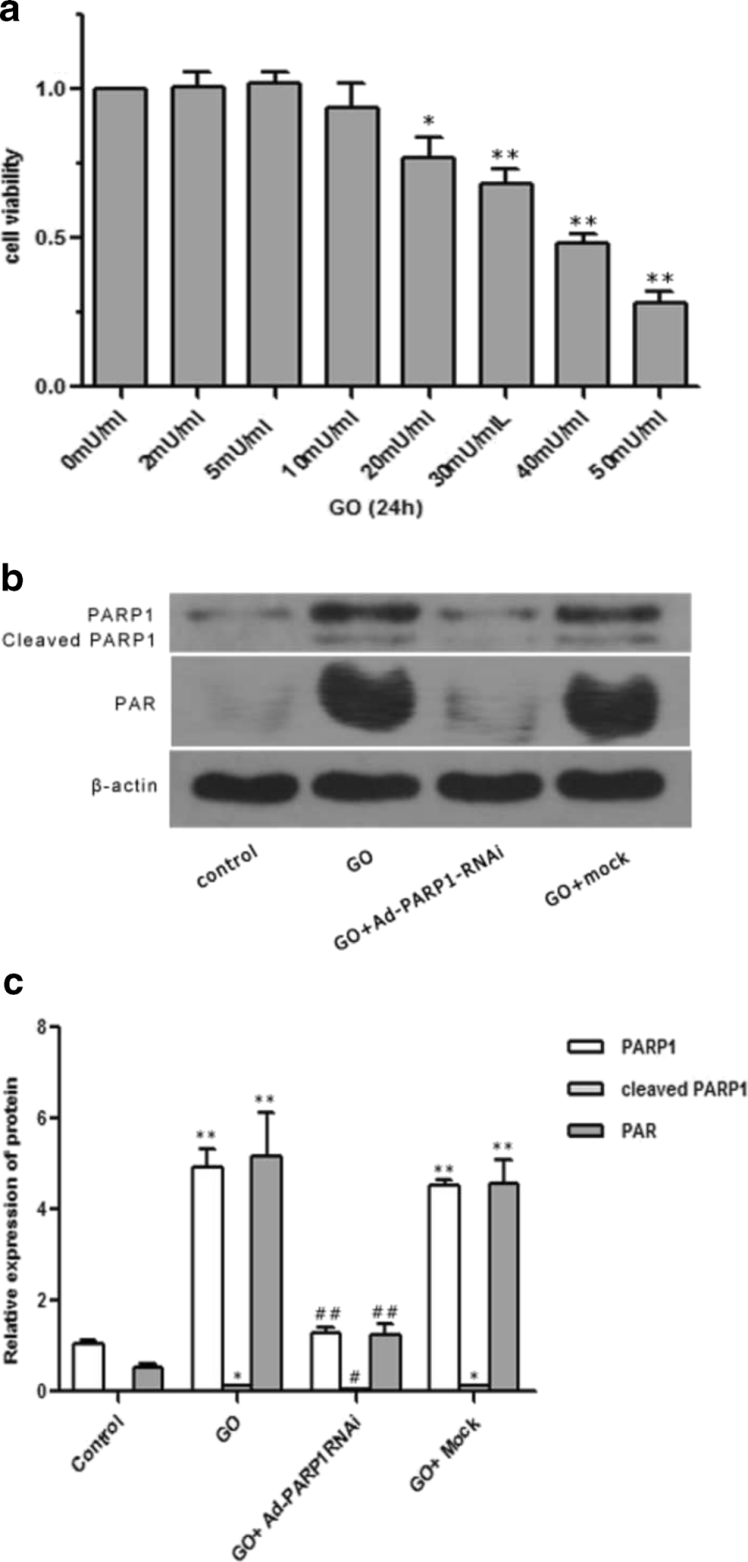
GO treatment induces PARP1 activation. The optimal concentration of GO treatment as determined by the CCK8 test. The control group was treated with 0 mU/ml GO. The cellular viability of groups that were exposed to over 10 mU/ml GO was all less than that of the control group, indicating that the growth of the MCs was suppressed when the GO concentration was greater than 10 mU/ml and that the appropriate concentration of GO was 20 mU/ml. **b**, **c** The relative protein levels of PARP1 and PAR in each group. The levels of PAR and PARP1 cleavage proteins (85 kDa) increased significantly in the GO group compared to the control group (*P* < 0.01), indicating that PARP1 was activated in MCs exposed to 20 mU/ml GO for 24 h. All of the *Y* axis values of the western blot assays represent the percentage of protein expression relative to β-actin. The data are presented as the mean ± SEM of three separate experiments. *P < 0.05 vs. the control group, **P < 0.01 vs. the control group, ##*P* < 0.01 vs. the GO group

Figure 3a shows that treatment with a lower concentration of GO slightly increased the viability of MCs, although the increase was not statistically significant. In contrast, GO concentrations over 10 mU/ml induced a statistically significant loss of viability in a concentration-dependent manner. We deduced that the concentration of GO required to induce oxidative damage in MCs was 20 mU/ml and that this results in a considerable reduction in viability compared to the 0 mU/ml treatment group (*P* < 0.05).

Western blotting revealed that, after treatment with 20 mU/ml GO for 24 h, PAR and PARP1 cleavage proteins (85 kDa) were significantly upregulated compared to the control group (Fig. 3b) (*P* < 0.01). In contrast, infection with the Ad-PARP1-RNAi virus before GO treatment downregulated the expression (*P* < 0.01).

### ROS Generation in MCs

**Fig. 4:**
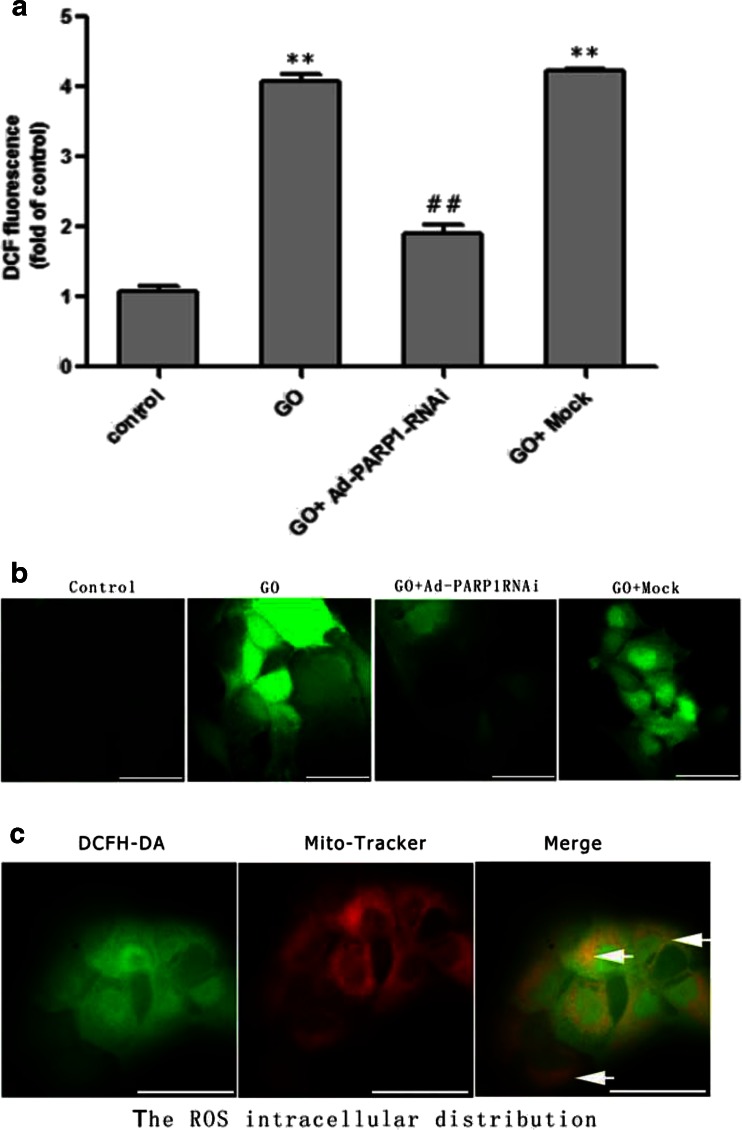
Detection of intracellular ROS levels. **a**, **b** The ROS accumulation (green fluorescent DCF) in MCs was measured using a fluorescence spectrometer (λex 490 nm, λem 515 nm) and by flow cytometry and fluorescence microscopy. **c** The distribution of intracellular ROS was detected by co-culturing the cells with MitoTracker (*red*) and DCFH-DA (*green*) and then observing them under a fluorescence microscope. **P < 0.01 vs. the control group, ##*P* < 0.01 vs. the GO group. Data are expressed as the mean + SEM (*n* = 3). *Scale bar* = 50 μm

The ROS accumulation and distribution in MCs were observed by flow cytometry and fluorescence microscopy, respectively. The results showed that exposure of MCs to 20 mU/ml GO for 24 h increased ROS generation approximately fourfold compared to the control group (Fig. 4a, b; *P* < 0.01). The ROS production of the GO + Ad-PARP1-RNAi group was significantly reduced compared to the GO group (*P* < 0.01). However, the GO + mock group did not exhibit any change in ROS generation when compared to the GO group (*P* > 0.05). Figure 4c shows that most of the DCFH-DA signals (green florescence) overlapped with the MitoTracker signals (red florescence). These findings suggested that the mitochondria were primarily responsible for the generation of ROS, rendering MCs sensitive to oxidative damage and that inhibition of PARP1 could counteract the GO-induced increase of intracellular ROS.

### MC Death from Exposure to GO

**Fig. 5:**
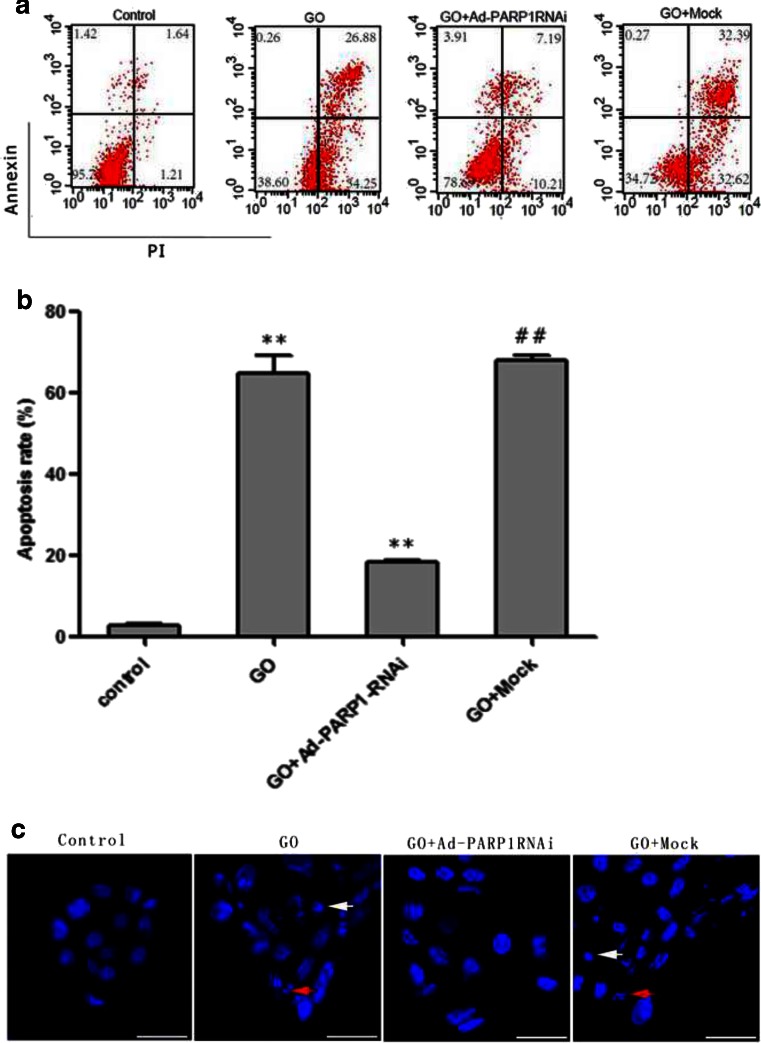
Detection of cell apoptosis. **a**, **b** The rate of apoptosis was determined by flow cytometry after annexin V-APC/PI staining. The *lower left quadrants* contain the viable cells (AV−/PI−), the *lower right quadrants* contain the early apoptotic cells (AV+/PI−), the *upper right quadrants* contain the late apoptotic cells (AV+/PI+), and the *upper left quadrants* contain the necrotic cells (AV−/PI+). **c** The morphology of MC nuclei was visualized by fluorescence microscopy after DAPI staining. Untreated MCs remained morphologically normal. Typical apoptotic changes such as nuclear condensation (*white arrows*) and nuclear fragmentation (*red arrows*) were observed in the GO group and in the GO + mock group. **P < 0.01 vs. the control group, ##*P* < 0.01 vs. the GO group. Data are expressed as the mean + SEM (*n* = 3). *Scale bar* = 50 μm

Figure 5a, b shows that exposure of MCs to 20 mU/ml GO for 24 h resulted in a significantly increased rate of apoptosis compared to the control group (*P* < 0.01). However, MCs in the GO + mock group did not exhibit any change in the rate of apoptosis compared to the GO group (*P* > 0.05).

DAPI staining revealed that, after exposure to GO for 24 h, the nuclei of the MCs were condensed and fragmented, suggesting that the cells were apoptotic. Fluorescence microscopy showed similar results. In the cells in the GO + Ad-PARP1-RNAi group, the characteristic apoptotic changes in the nuclei were much less pronounced (Fig. 5c). The findings were consistent with the results of FACS.

### PARP1-Modulated MC Death

**Fig. 6:**
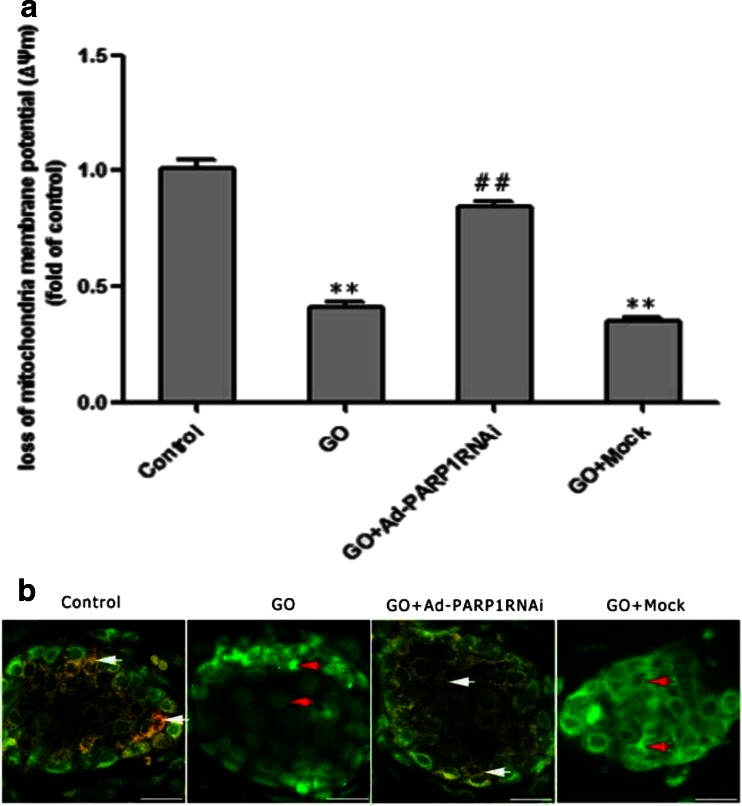
PARP1 RNAi ameliorates the loss of mitochondrial membrane potential. Changes in ΔΨm between groups of MCs were detected using a multifunctional microplate reader (**a**) and a fluorescence microscope (**b**). **a** The fluorescence signals (Ex = 485 nm/Em = 590 nm) of ΔΨm in MCs were monitored on a multifunctional microplate reader after they were loaded with MitoTracker. **b** Upon analysis by fluorescence microscopy, the healthy cells present orange florescence (*white arrows*), and the cells with a decreased ΔΨm emitted only green fluorescence (*red arrows*). Data are expressed as the mean + SEM (*n* = 3). **P < 0.01 vs. the control group, ##*P* < 0.01 vs. the GO group. *Scale bar* = 50 μm

To assess mitochondrial damage in MCs after exposure to GO, we measured ΔΨm in MCs by fluorescence detection using a microplate reader and under a microscope. Concomitant with the increase in mitochondrial ROS levels, MC ΔΨm sharply declined in the GO and GO + mock groups compared to the control group (Fig. 6a; *P* < 0.01), and the ΔΨm increased significantly in the GO + Ad-PARP1-RNAi group (*P* < 0.01). Under a fluorescence microscope, MCs in the GO and GO + mock groups presented green fluorescence, which is indicative of apoptotic cells. On the contrary, MCs in the GO + Ad-PARP1-RNAi and control groups displayed a large amount of orange fluorescence, which is indicative of healthy cells (Fig. 6b). Data obtained from the fluorescence microplate reader showed similar results (Fig. 6a). These findings suggested the PARP1 could modulate the GO-induced apoptosis of MCs via mitochondria, but that inhibition of PARP expression could protect mitochondria from dysfunction.

### Relocation of Bax and Cytochrome C

**Fig. 7:**
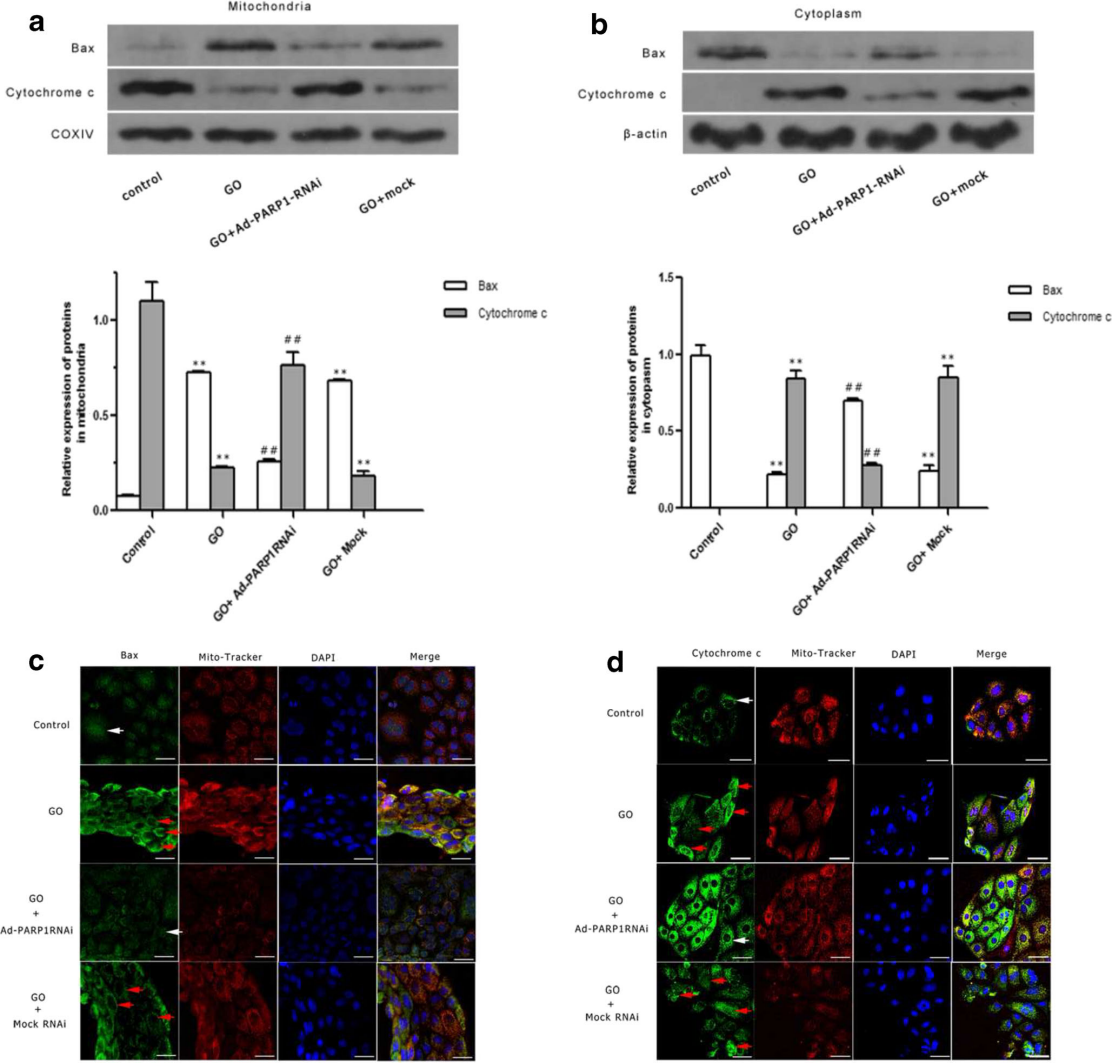
Relocation of Bax and CytC. **a**, **b** Western blot analysis showed the relative expression of Bax and cytochrome c in the mitochondria and cytoplasm, respectively. **c**, **d** The immunofluorescence of Bax, cytochrome c, and MitoTracker and their subcellular distribution during the GO-induced apoptosis of MCs were detected simultaneously by confocal microscopy. *Green fluorescence* represents Bax and cytochrome c and *red fluorescence* is indicative of MitoTracker. *White arrows* show the distribution of Bax and cytochrome c in the control and GO + Ad-PARP1RNAi group cells (**c**, **d**). *Red arrows* indicate the redistribution of Bax and cytochrome c in the GO and GO + mock group cells. The results are given as the mean of three separate experiments. *Scale bar* = 50 μm

Western blotting and immunofluorescence were performed to investigate the possible mechanism of MC death after ΔΨm loss and to elucidate the role of PARP1 in the process. Western blotting showed that the expression of Bax in the mitochondria was increased in the GO group, and, as a result, Bax protein levels were reduced in the cytosol compared to control cells. Pretransfection with Ad-PARP1-RNAi could significantly counteract this change (Fig. 7a; *P* < 0.01). There was no difference in protein relocation between the GO + mock and GO groups (*P* > 0.05). The relocation pattern of cytochrome c in MCs was opposite to that of Bax (Fig. 7b).

Consistent with the results of western blotting, fluorescence microscopy showed that Bax (green fluorescence) was located in the cytosol and separated from the MitoTracker (red fluorescence) and that cytochrome c fully overlapped with mitochondria in control cells, presenting orange fluorescence (Fig. 7c, d). Conversely, Bax gradually migrated into mitochondria after GO treatment and partially overlapped with red MitoTracker, indicating they colocalized in the mitochondria (Fig. 7c). Moreover, cytochrome c was scattered in the cytosol (Fig. 7d). However, preinfection with Ad-PARP1-RNAi could clearly counteract these changes. In the GO + mock group, no such changes were observed.

### Effect of z-VAD-fmk on the Death of MCs

Activation of the classic caspase-dependent pathway is the core mechanism that leads to apoptotic cell death. Release of cytochrome c from the mitochondria to the cytosol is essential for the induction of apoptosis [29]. The finding that inhibition of PARP1 could prevent GO-induced cell death prompted us to examine the correlation between caspase-3 activation and activated PARP1.

**Fig. 8:**
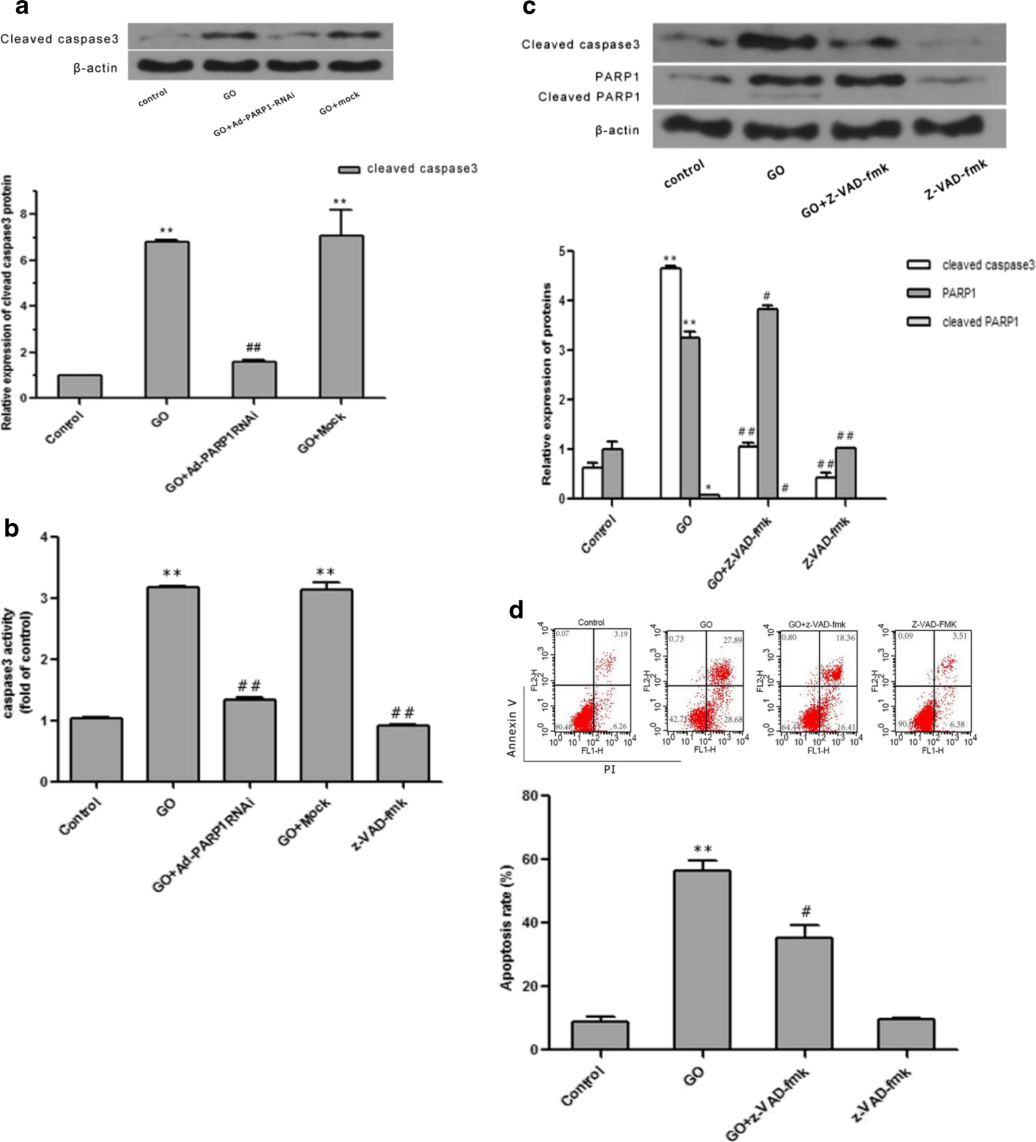
PARP1-induced MC death is partly caspase-dependent. **a** Western blot analysis showed the relative expression of cleaved caspase-3 in each group. **b** The activity of caspase-3 following treatment of each group was measured using a multifunctional microplate reader. **c** The change in the amounts of cleaved caspase-3 and PARP1 expression in MCs was detected by western blotting. **d** Differences in the rate of MC apoptosis were determined by flow cytometry after annexin V-APC/PI staining. Data are expressed as the mean + SEM (*n* = 3). **P < 0.01 vs. the control group, #*P* < 0.05 vs. the GO group, ##*P* < 0.01 vs. the GO group

Figure 8a shows that the amount of cleaved caspase-3 was significantly higher in the GO group than in the control group (*P* < 0.01) and that caspase-3 cleavage was significantly lower in the GO + Ad-PARP1-RNAi group (*P* < 0.01) compared to the GO group. This change in the pattern of cleaved caspase-3 was similar to that observed with PAR (Fig. 3b) which is an indicator of the activity of PARP1.

We then pretreated MCs with 50 μmol/l z-VAD-fmk for 30 min before GO treatment and measured caspase-3 activity using Ac-DEVD-AFC, a caspase-3-specific substrate. Figure 8b shows that caspase-3 activity sharply increased in the GO group compared to the control group (*P* < 0.01), while in both the GO + Ad-PARP1-RNAi and GO + z-VAD-fmk groups, caspase-3 activity apparently decreased compared to the GO group (*P* < 0.01). There were no differences in caspase-3 activity between the GO + mock and GO groups (*P* > 0.05). These findings indicated that caspase-3 was activated by GO treatment and that its activity could be abolished by z-VAD-fmk or by inhibiting PARP1.

Western blotting showed that the levels of both cleaved caspase-3 and cleaved PARP1 were reduced in the GO + z-VAD-fmk group compared to the GO group (Fig. 8c; *P* < 0.05) but that full-length PARP1 was not inhibited.

Flow cytometry revealed that the apoptosis of MCs was decreased to some extent in the GO + z-VAD-fmk group compared to the GO group (Fig. 8d; *P* < 0.05). However, the rate of MC apoptosis in the GO + z-VAD-fmk group (34.77 %) was higher than that (17.4 %) in the GO + Ad-PARP1-RNAi group (Fig. 5a).

## Discussion

Maintenance of endocochlear potential is essential for normal hearing and depends primarily on the pumping mechanism of the marginal cells of the cochlear lateral wall. A wide array of pathological and physiological changes induced by aging, stress, ischemia, noise, and ototoxic agents can cause irreversible damage to marginal cells, leading to excessive generation of ROS and eventually to auditory impairment. PARP1, a molecular sensor of DNA damage [30], is activated when DNA is damaged and is thought to play a role in a variety of diseases, such as diabetes [6, 22], arthritis [23], Parkinson’s and Alzheimer’s diseases, cerebral ischemia, traumatic brain injury [24], and some malignancies such as ovarian carcinoma [25]. Regarding the inner ear, most studies about the effect of PARP1 have been conducted in the outer hair cells and a few have been carried out in spiral ganglion neurons. In fact, little research effort has been directed at the role of PARP1 in MCs. However, at present, the role that PARP1 plays in different inner cells remains controversial. Shi [31] demonstrated that PARP1 was activated in some marginal cells and in some vessels of the spiral ligaments and that activated PARP1 could stimulate the expression of P-selectin and platelet-endothelial cell-adhesion molecule-1 in the vessels of the spiral ligament after exposure to loud noise, thereby triggering inflammatory events. Although Jiang [32] found that PARP1 was decreased in a mouse model of progressive kanamycin-induced hair cell loss, they failed to detect the 89-kDa PARP1 fragment derived from caspase and markers of classic apoptotic pathways (cytochrome c, caspase-9, caspase-3, JNK, and TUNEL). Thus, the role of PARP1 in the oxidative damage of MCs remains poorly understood. In this study, we established an in vitro cellular oxidative stress model using GO and attempted to explore the role that PARP1 plays in the oxidative damage of MCs.

In this study, the treatment of MCs with 20 μM/ml GO induced an apparent loss of cell viability. Meanwhile, PARP1 expression and activity were significantly enhanced in the GO-treated group compared to the control group (Fig. 3; *P* < 0.01), as was ROS generation and MC apoptosis (Figs. 4 and 5; *P* < 0.01). DAPI staining also revealed typical apoptotic changes, such as nuclear shrinkage and fragmentation. PARP1 activity was counteracted by preinfection with Ad-PARP1-RNAi, and this was accompanied by the downregulation of ROS generation and MC apoptosis. These results suggest that ROS generation and MC death could be regulated by activated PARP1 and raise the question as to how PARP1 regulates the death of MCs.

Excessive ROS generation is generally followed by three changes that lead to cell apoptosis: depolarization of the inner mitochondrial membrane, release of Bcl-2 family proteins from the mitochondria, and the opening of the permeability transition pore (PTP) [33–35]. Of these, loss of mitochondrial membrane potential (ΔΨm) is believed to be a crucial factor in the apoptotic pathway. In most cells, excessive ROS are produced by the mitochondria, which are easy targets of ROS attack [36]. This study showed that the intense green DCFH-DA signals overlapped with the red MitoTracker signals in GO-treated MCs, suggesting that GO could induce excessive ROS generation and that mitochondria are responsible for the local generation of most ROS. Additionally, LSCM and western blotting showed that, in GO-treated MCs, the decrease in ΔΨm was accompanied by the recruitment of Bax from the cytosol to the mitochondria and that this was followed by relocation of cytochrome c from the mitochondria to the cytosol. Furthermore, decreased PARP1 protein levels inhibited the relocation of a series of death-associated proteins and prevented the death of MCs. Our study suggested that strong PARP activation might transmit a death signal to the mitochondria, thereby causing depolarization of the mitochondrial transmembrane potential and the relocation of Bax, a member of the anti-apoptotic protein family, leading to the opening of high-permeability pores and the release of cytochrome c from the mitochondria to the cytoplasm. Our results demonstrated that PARP1 activation could trigger and regulate MC death via a mitochondria-mediated pathway.

Our findings raise the question as to how PARP1 siRNA downregulates ROS generation in MCs and protects mitochondria from oxidative damage. Previous reports showed that oxidant-induced functional changes in the mitochondria are related to PARP1 activation rather than to the direct effect of the oxidant on the mitochondria [37]. A number of studies have shown that the poly-ADP-ribosylation of nuclear proteins is involved in DNA repair or transcriptional regulation [7, 38]. Less is known about how PARP(s) work on mitochondrial proteins. Many studies have demonstrated that, in mitochondria, poly-ADP-ribosylation occurred [39–41], which may be involved in mitochondrial function. Lai [42] believed that mitochondrial PARP1 could ADP-ribosylate many mitochondrial proteins (VDAC1, mitofilin, cytochrome c reductase core protein, COX subunit Va, etc.) at the transcriptional level, thereby resulting in the dysfunction of the respiratory chain. The dysfunction of the respiratory chain could lead to the generation of excessive ROS, which cannot be removed in time, eventually causing functional impairment of the mitochondria [43]. We found that some PARP1 was localized to the mitochondria (Fig. 2a), and this led us to believe that mitochondrial PARP1 might be involved in the regulation of mitochondria-mediated MC death induced by GO.

Activation of the caspase cascade is intimately correlated to mitochondrial oxidative dysfunction. Once cytochrome c releases from damaged mitochondria and enters the cytosol, the caspase cascade will be activated. Cytochrome c plays a primary role in the formation of the apoptosome complex by activating the binding of procaspase-9 to Apaf-1 [44]. The complex then activates caspase-3, leading to cleavage of PARP1 and caspase-dependent apoptosis [20, 45]. To further clarify the presence of the classic apoptotic cascade in GO-induced MC apoptosis and the relationship between PARP1 and caspase-3, we detected the cleavage of caspase-3, an indicator of caspase-3 activity. The results indicated that PARP1 siRNA effectively prevented caspase-3 activation in MCs compared to MCs treated with GO alone (Fig. 8a; *P* < 0.01), suggesting that caspase activation was a downstream event of PARP activation.

To further understand whether the caspase-dependent pathway was solely responsible for PARP-dependent apoptosis in MCs, we pretreated the cells with the pan-caspase inhibitor z-VAD-fmk before GO exposure. We found that the cleavage and activity of caspase-3 were much lower in the z-VAD-fmk-pretreated group than in the GO-treated group. In addition, the z-VAD-fmk-pretreated cells showed a decrease in cleaved PARP1, a product of full-length PARP1 cleavage. The study illustrated that z-VAD-fmk effectively inhibited caspase-3 activity and decreased the PARP1 cleavage fragments as opposed to full-length PARP1 in MCs. In agreement with these results, flow cytometry showed that the apoptosis of MCs was suppressed, although not completely, by z-VAD-fmk pretreatment (Fig. 7c; *P* < 0.05) and that the suppression was less than when PARP1 expression was inhibited (Fig. 5a). We conclude that the caspase-associated apoptotic pathway is not the sole pathway implicated in PARP1-dependent MC death induced by GO. There may be other mechanisms in addition to the typical caspase-dependent pathway that function in MCs. This notion is different from the conclusion drawn from a previous study suggesting that z-VAD-fmk could completely block the NGF/p75ngfr/ceramide-induced apoptosis of epithelial cells and neurons in otocysts and the cochleovestibular ganglia [46]. A more recent study found that z-VAD-fmk significantly reduced the number of annexin V-positive renal cancer cells and that this apoptosis was considered to be caspase-dependent [47]. However, Jiang et al. failed to detect the markers of classic apoptotic pathways (cytochrome c, caspase-9, caspase-3, JNK, and TUNEL) in a mouse model of progressive kanamycin-induced hair cell loss [32].

Based on our results, we concluded the following: (1) Treatment of MCs with GO could mimic oxidative damage to MCs and that GO could considerably suppress MC activity and induce massive ROS generation and apoptosis in MCs. (2) Mitochondria are responsible for the generation of most local ROS in MCs and are vulnerable to ROS attack induced by GO. (3) GO treatment induces the activation of PARP1, leading to a decreased ΔΨm via mitochondrial mediation, and facilitates the relocation of the molecules in the downstream pathway. Mitochondrial PAR might take part in the regulation of mitochondria-mediated MC death. (4) PARP1 plays a pivotal role in GO-induced MC death that is, at least in part, via the caspase-3 pathway. (5) In addition to the caspase-dependent pathway, there might be other apoptotic pathways involved in PARP1-dependent GO-induced MC death.
